# More than one antibody of individual B cells revealed by single-cell immune profiling

**DOI:** 10.1038/s41421-019-0137-3

**Published:** 2019-12-10

**Authors:** Zhan Shi, Qingyang Zhang, Huige Yan, Ying Yang, Pingzhang Wang, Yixiao Zhang, Zhenling Deng, Meng Yu, Wenjing Zhou, Qianqian Wang, Xi Yang, Xiaoning Mo, Chi Zhang, Jing Huang, Hui Dai, Baofa Sun, Yongliang Zhao, Liang Zhang, Yun-Gui Yang, Xiaoyan Qiu

**Affiliations:** 1 Department of Immunology, School of Basic Medical Sciences, Peking University, Beijing, 100191 China; 2 NHC Key Laboratory of Medical Immunology, Peking University, Beijing, 100191 China; 3 Key Laboratory of Molecular Immunology, Chinese Academy of Medical Sciences, Beijing, 100191 China; 4 CAS Key Laboratory of Genomic and Precision Medicine, Collaborative Innovation Center of Genetics and Development, Beijing Institute of Genomics, Chinese Academy of Sciences, Beijing, 100101 China; 5 Sino-Danish College, University of Chinese Academy of Sciences, Beijing, 101408 China; 6 Institute of Stem Cell and Regeneration, Chinese Academy of Sciences, Beijing, 100101 China; 7 Key Laboratory of Biochip Technology, Biotech and Health Centre, Shenzhen Research Institute of City University of Hong Kong, Shenzhen, 518057 China; 8 Department of Biomedical Science, Jockey Club College of Veterinary Medicine and Life Sciences, City University of Hong Kong, 83 Tat Chee Avenue, Kowloon, Hong Kong China; 9 College of Future Technology, University of Chinese Academy of Sciences, Beijing, 100049 China

**Keywords:** Immunology, Innate immunity

## Abstract

Antibodies have a common structure consisting of two identical heavy (H) and two identical light (L) chains. It is widely accepted that a single mature B cell produces a single antibody through restricted synthesis of only one V_H_DJ_H_ (encoding the H-chain variable region) and one V_L_J_L_ (encoding the L-chain variable region) via recombination. Naive B cells undergo class-switch recombination (CSR) from initially producing membrane-bound IgM and IgD to expressing more effective membrane-bound IgG, IgA, or IgE when encountering antigens. To ensure the “one cell — one antibody” paradigm, only the constant region of the H chain is replaced during CSR, while the rearranged V_H_DJ_H_ pattern and the L chain are kept unchanged. To define those long-standing classical concepts at the single-cell transcriptome level, we applied the Chromium Single-Cell Immune Profiling Solution and Sanger sequencing to evaluate the Ig transcriptome repertoires of single B cells. Consistent with the “one cell — one antibody” rule, most of the B cells showed one V(D)J recombination pattern. Intriguingly, however, two or more V_H_DJ_H_ or V_L_J_L_ recombination patterns of IgH chain or IgL chain were also observed in hundreds to thousands of single B cells. Moreover, each Ig class showed unique V_H_DJ_H_ recombination pattern in a single B-cell expressing multiple Ig classes. Together, our findings reveal an unprecedented presence of multi-Ig specificity in some single B cells, implying regulation of Ig gene rearrangement and class switching that differs from the classical mechanisms of both the “one cell — one antibody” rule and CSR.

## Introduction

Immunoglobulins (Igs), also called antibodies, are composed of four peptide chains (two identical heavy (H) and two identical light (L) chains) and produced by B lymphocytes. Igs are capable of recognizing almost every kind of antigen, and this ability is primarily attributed to the extreme diversity and specificity of their antigen-binding portions, known as variable (V) regions. The diversity of IgV regions takes shape through a process of gene rearrangement during B-cell development, which creates functional IgV(D)J transcripts from multiple copies of the Variable (V), Diversity (D), and Joining (J) gene segments at the genomic level^1–3^. The chromosomal region that encodes the IgH chain consists of multiple copies of these V, D, and J segments, while the light-chain loci contain two types of genes, κ and λ, which have V or J segments but lack D segments^2,4^. To produce functional Igs, the separate V, D, and J segments must be rearranged into V_H_DJ_H_ and V_L_J_L_ recombinants at the corresponding chromosomes to form the variable regions of the H chain and L chain, respectively^3,5^. According to the current clonal selection theory^5–7^, the process of producing V_H_DJ_H_ and V_L_J_L_ recombinants includes (1) randomly selecting each segment of V, D, and J for the H chain (or V and J for the L chain); (2) introducing double-strand breaks (DSBs) adjacent to each segment by the rearrangement activation genes recombination activating 1 and 2 (RAG1 and RAG2)^3,8^; (3) deleting the intervening DNA^7,9^; and (4) ligating the remaining segments. To ensure the “one B lymphocyte — one antibody” paradigm, rearrangement of the H or L chain is allowed to occur on only one chromosome (allelic exclusion)^7,9,10^. Similarly, V_L_J_L_ rearrangement in each single B cell occurs in only one type of either the κ or λ chain (isotype exclusion)^4^. So far, this clonal selection theory has been widely accepted, and meanwhile, the “one cell — one antibody” rule has also been supported by early surface membrane analysis of B lymphocytes using Ig-allotype-specific antisera^11,12^ and further confirmed by later monoclonal antibody-producing hybridoma cells^13–15^. However, whether this concept can be reproduced at the level of a single B-cell transcriptome remains unknown.

A diverse repertoire of antibodies contributes to immune recognition and defense against the threats of a vast number of potential pathogens. Igs are initially expressed as IgM in immature B cells^16^, or IgM and IgD with identical antigen specificity and concurrently low affinity in mature naive B cells^17^. Upon encountering antigens, the naive B cells undergo somatic hypermutation (SHM)^18^ in the V exons and affinity-based selection in the germinal centers (GCs)^19^. Clones with mutated V exons that encode higher-affinity Igs gain an advantage in the competition for limited help from cognate T cells, leading to antibody affinity maturation^20^. Subsequently, to generate a more efficient Ig class, the activated B cells further undergo class-switch recombination (CSR), in which exons encoding the default Cμ constant region of the IgH chain are excised and replaced with a new constant region gene segment (referred to as “C_H_ genes”, e.g., Cγ, Cα, or Cε)^21–23^, leading to a switch in Ig expression from IgM to IgG, IgA, or IgE, whereas the rearranged V(D)J patterns remain largely unchanged, except that SHM sometimes occurs in the V segment^24,25^. Thus, the activated B cells differentiate into plasma cells or memory B cells, changing their Ig expression from low-affinity IgM to high-affinity IgG, IgA, or IgE. Advances in single-cell sequencing techniques now allow a clear delineation of the CSR process at the single-cell transcriptome level, which has been lacking to date.

In this study, we evaluated the Ig transcriptome repertoires of individual B cells by immune profiling using single-cell RNA-seq (scRNA-seq) combined with Sanger sequencing. Intriguingly, in addition to the classical “one B cell — one antibody” profile in most B cells, two or more V_H_DJ_H_ or V_L_J_L_ recombination patterns of the IgH chain or L chain were also observed in hundreds to thousands of single B cells, including naive B cells, memory B cells, and plasma cells. Moreover, the κ chain and λ chain were frequently co-expressed in a single B cell. Importantly, several classes of IgH chain were expressed synchronously in a single B cell and showed distinct VDJ patterns. Taken together, our findings suggest the existence of novel patterns of Ig gene rearrangement and class switching in a single B cell.

## Results

### Two or more V_H_DJ_H_ or V_L_J_L_ recombination patterns present in some single B cells

**Fig. 1:**
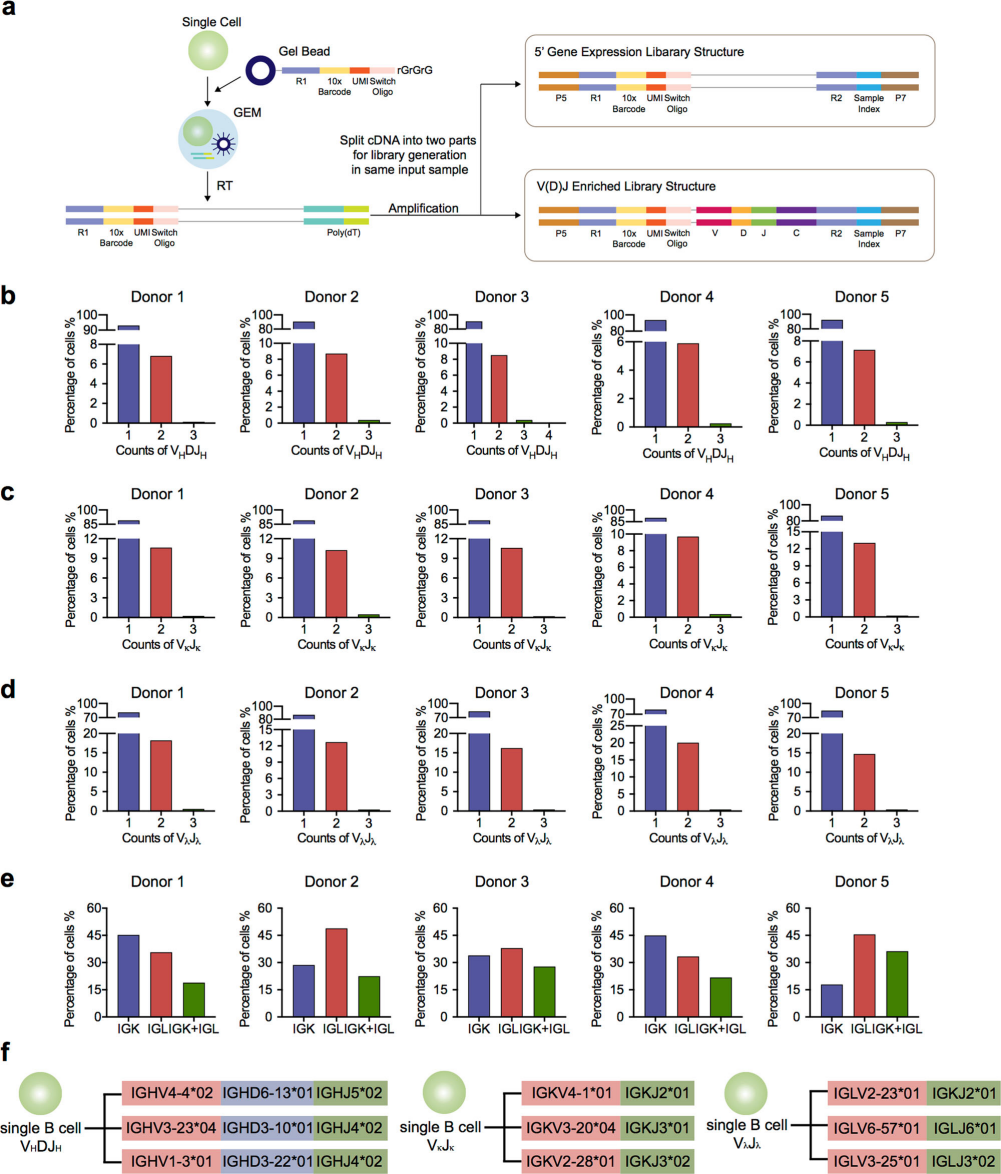
Single-cell immune profiling revealed multiple V_H_DJ_H_ or V_L_J_L_ recombination patterns in single B cells. **a** Schematic illustration of the Chromium Single-Cell Immune Profiling Solution. Single-cell 5′ GEX and V(D)J enrichment libraries can be generated from the same B cell. **b**–**d** Percentages of single B cells expressing one, two, or three V_H_DJ_H_ segments (**b**), V_κ_J_κ_ segments (**c**), and V_λ_J_λ_ segments (**d**) in five donors. Single B cells expressing VDJ recombination patterns, donor 1, *n* = 5037; donor 2, *n* = 3112; donor 3, *n* = 3708; donor 4, *n* *=* 2253; donor 5, *n* = 3603. Single B cells expressing V_κ_D_κ_ recombination patterns, donor 1, *n* = 3396; donor 2, *n* = 2641; donor 3, *n* = 2364; donor 4, *n* = 1586; donor 5, *n* = 2371. Single B cells expressing V_λ_J_λ_ recombination patterns, donor 1, *n* = 2572; donor 2, *n* = 1686; donor 3, *n* = 2280; donor 4, *n* = 1221; donor 5, *n* = 1821. **e** Percentages of single B cells expressing only Igκ or Igλ or expressing Igκ and Igλ in five donors. Donor 1, *n* = 5366; donor 2, *n* = 3356; donor 3, *n* = 3843; donor 4, *n* = 2483; donor 5, *n* = 3745. **f** Examples of three V_H_DJ_H_ or V_κ_J_κ_ or V_λ_J_λ_ recombination patterns found in single B cells

To explore the Ig repertoire in single B cells, we first sorted CD19^+^ B cells from the peripheral blood of five healthy donors (donors 1–5) by flow cytometry and employed scRNA-seq and V(D)J-seq using the Chromium Single-Cell Immune Profiling Solution (Fig. 1a)^26,27^. The 5′ gene expression (GEX) library and V(D)J immune repertoires (Ig transcripts of all five classes of IgH chains and two types of IgL chains) in a total of 18,899 CD19^+^ cells sorted from five donors were acquired in the same input sample. Approximately, 90% of CD19^+^ cells from the V(D)J enrichment assays were also detected by 5′ GEX analysis, linking both cell type and immune repertoires in the same cells. We analyzed the number of V_H_DJ_H_ and V_L_J_L_ recombination patterns in each individual B cell and found that most single B cells displayed one V_H_DJ_H_ or V_L_J_L_ (either V_κ_J_κ_ or V_λ_J_λ_) recombination, consistent with the classical concepts. Unexpectedly, some single B cells from all five donors also displayed two V_H_DJ_H_ (5.90–8.71%) or V_κ_J_κ_ (9.71–13.07%) or V_λ_J_λ_ (12.69–20.07%) recombination patterns, or even more than two V_H_DJ_H_ (0.16–0.42%), V_κ_J_κ_ (0.21–0.49%) or V_λ_J_λ_ (0.30–0.58%) recombination patterns (Fig. 1b-d; Supplementary information, Tables S1–5). Moreover, 18.02–28.64% single B cells co-expressed the V_κ_J_κ_ and V_λ_J_λ_ recombination patterns (Fig. 1e). Examples of different V(D)J recombination patterns in single B cells are shown in Fig. 1f, and the sequences of the V_H_DJ_H_, V_κ_J_κ_, and V_λ_J_λ_ recombination patterns in each single B cell are listed in Supplementary information, Tables S1–5.

Given the restriction of the 10× Genomics technology, there may be some transcribed IgV, IgD, and IgJ sequences failed to be assembled to intact V–D–J or V–J sequences. Therefore, to explore if there are more V(D)J recombination patterns in some single B cells than we have detected, we further analyzed the transcript counts of IgV segments, which are much longer and more conserved than IgD and IgJ. As expected, more single B cells were found to express multiple IgV segments, with a much higher proportion expressing more than one immunoglobulin heavy-chain variable segments (IGHV, 49.73%, 2886/5803) (Supplementary information, Fig. S1a), Igκ chain variable segments (IGKV, 62.45%, 3138/5025) (Supplementary information, Fig. S1b) and Ig λ chain variable segments (IGLV, 73.13%, 3756/5136) (Supplementary information, Fig. S1c). The percentage of cells expressing multiple V segments in plasma cells was much higher than those in naive and memory B cells (Supplementary information, Fig. S1d–f). Similarly, 61.12% of single B cells and 93.33% of plasma cells co-expressed V_κ_J_κ_ and V_λ_J_λ_ recombinant transcripts (Supplementary information, Fig. S1g, h). We also observed the transcripts of two or more Ig classes in 69.98% of single B cells (Supplementary information, Fig. S1i, j), among which some B cells even expressed all five Ig classes.

We then analyzed the scRNA-seq data of peripheral blood mononuclear cells (PBMCs) from a healthy donor in the Gene Expression Omnibus (GEO) database (GEO: GSE111360) and detected multiple V_H_DJ_H_ (10.63%), V_κ_J_κ_ (11.61%), and V_λ_J_λ_ (18.95%) recombination patterns in single B cells (Supplementary information, Fig. S2a–c and Table S6). Moreover, 13.08% of single B cells co-expressed V_κ_J_κ_ and V_λ_J_λ_ recombinant transcripts (Supplementary information, Fig. S2d). Analysis of the data retrieved from the single-cell data website of 10× Genomics (https://support.10xgenomics.com/single-cell-vdj/datasets/2.2.0/vdj_v1_hs_pbmc_b) also revealed similar results (Supplementary information, Fig. S2e–g and Table S7). Overall, these findings suggest that polyclonal Igs are produced in some single B cells and escape the mechanisms of “allelic exclusion” and “isotype exclusion”.

### Multiple Ig classes with different VDJ recombination patterns expressed in single B cells

Upon encountering antigens, naive B cells primarily expressing the transcripts of the μ and δ chains will undergo CSR and change the expression patterns of the IgH constant region (C_H_) exons from Cμ to Cγ, Cα, or Cε, while the recombined VDJ is kept unchanged^22,23^. Thus, theoretically, a single memory B cell or plasma cell transcribes one class of IgH chain, perhaps the γ chain or α chain or ε chain, an IgH derived from a single B cell is supposed to display the same V_H_DJ_H_ pattern.

**Fig. 2:**
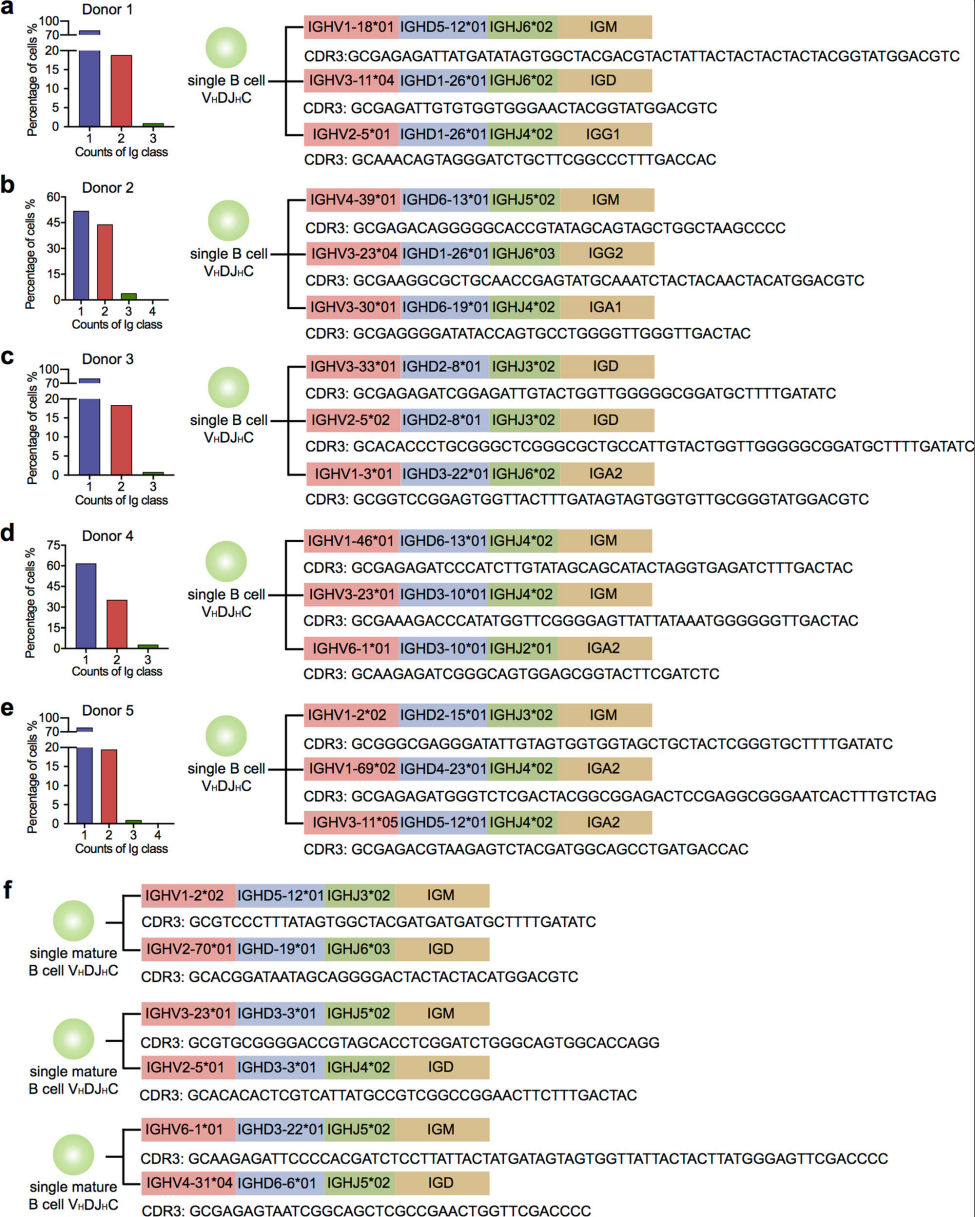
Multiple Ig classes in single B cells with unique V_H_DJ_H_ patterns. **a**–**e** Proportions of single B cells expressing one, two, or three Ig classes (left panel), and examples of the V_H_DJ_H_ recombination patterns and CDR3 sequences in single cells expressing several Ig classes (right panel). Donor 1, *n* = 5181 (**a**); donor 2, *n* = 3238 (**b**); donor 3, *n* = 3761 (**c**); donor 4, *n* = 2879 (**d**); donor 5, *n* = 3668 (**e**). **f** Single mature B cells expressing IgM and IgD with different productive V_H_DJ_H_ recombination patterns. IGHM immunoglobulin heavy constant μ chain, IGHD immunoglobulin heavy constant δ chain, IGHG immunoglobulin heavy constant γ chain, IGHA immunoglobulin heavy constant α chain, IGHE immunoglobulin heavy constant ε chain

In this study, we analyzed the expressing frequency of different Ig classes in single B cells. We found that among 18789 single B cells (five donors), 18.37–44.01% of single B cells showed expression of two Ig classes, and that moreover, 0.93–3.98% expressed three Ig classes (Fig. 2a–e, left panel). This finding led us to analyze the VDJ recombination patterns among different Ig classes in single B cells expressing more than one Ig classes. Surprisingly, each individual Ig class showed unique mono- or oligo-VDJ patterns, and few identical VDJ recombination patterns were shared among different Ig classes in any single B cell (Fig. 2a–e, right panel, Supplementary information, Tables S1–5). Importantly, it has been widely accepted that mature B cells express IgM and IgD with the same V_H_DJ_H_ recombination pattern, although without evidence from single B cells. We analyzed the V_H_DJ_H_ recombination patterns in 456 single B cells co-expressing IgM and IgD, and found that IgM and IgD in a single B cell showed different productive V_H_DJ_H_ recombination patterns (Fig. 2f).

Moreover, the results of scRNA-seq analysis of PBMCs from a healthy donor in the GEO database (GEO: GSE111360) also supported the presence of multiple Ig classes with different VDJ recombination patterns in single B cells (Supplementary information, Fig. S2h and Tables S6, S7). These results suggest that the previous classical CSR theory might require complementation or correction.

### Multi-specificity in single memory B cells

**Fig. 3:**
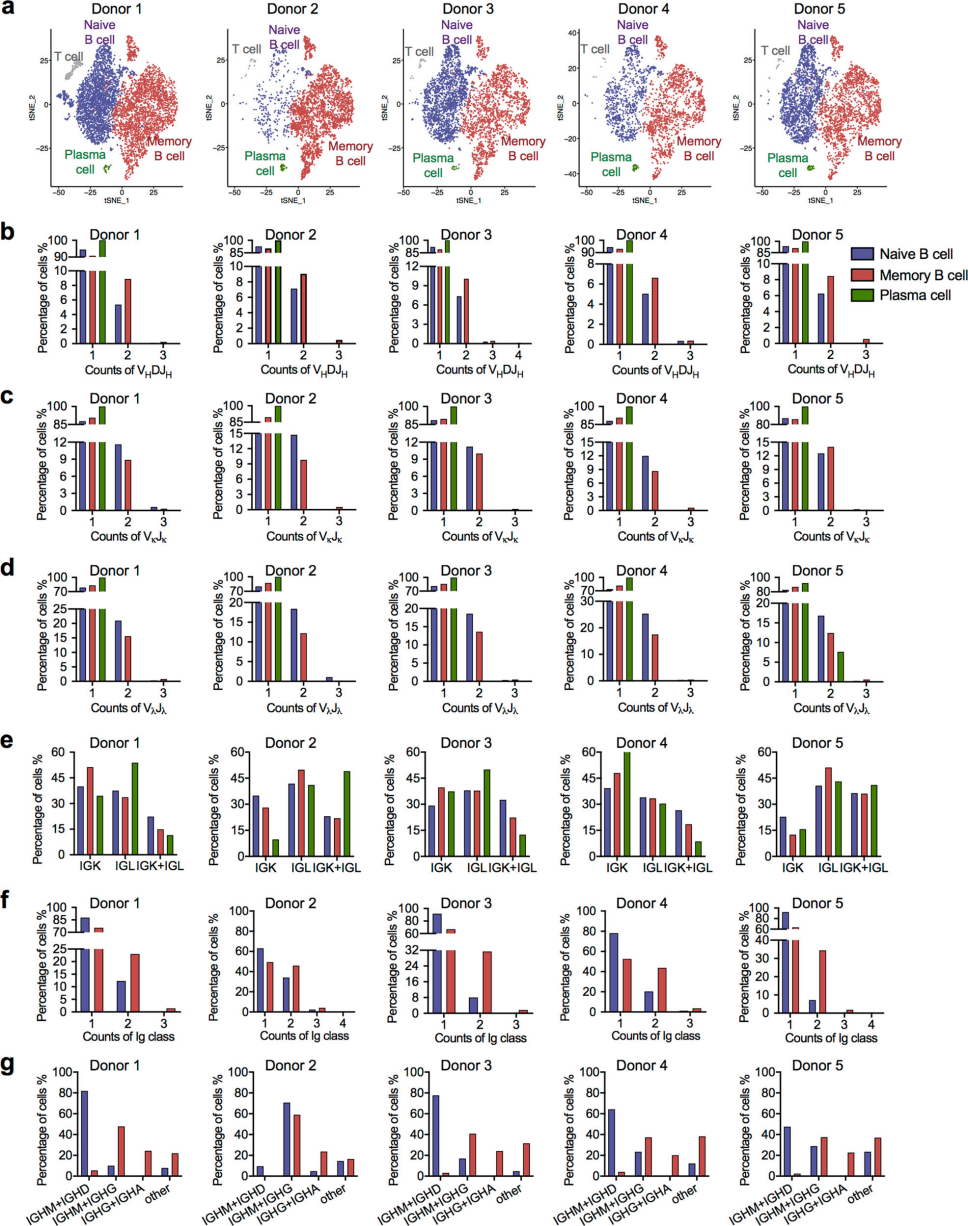
Single naive B cells and memory B cells display multi-specificity V(D)J recombination patterns. **a** t-SNE plot of 5′ single-cell RNA (scRNA)-seq from five healthy donors’ peripheral blood B cells. Donor 1: naive B cells, *n* = 2716; memory B cells, *n* = 2296; plasma cells, *n* = 25. Donor 2: naive B cells, *n* = 350; memory B cells, *n* = 2711; plasma cells, *n* = 51. Donor 3: naive B cells, *n* = 2074; memory B cells, *n* = 1610; plasma cells, *n* = 24. Donor 4: naive B cells, *n* = 852; memory B cells, *n* = 1357; plasma cells, *n* = 46. Donor 5: naive B cells, *n* = 1946; memory B cells, *n* = 1607; plasma cells, *n* = 50. **b**–**d** Proportions of single naive B cells, memory B cells, and plasma cells expressing one, two, or three V_H_DJ_H_ segments (**b**), V_κ_J_κ_ segments (**c**) and V_λ_J_λ_ segments (**d**) in five donors. **e** Proportions of single naive B cells, plasma cells, and memory B cells expressing only Igκ or Igλ or expressing Igκ and Igλ in five donors. **f** Proportions of single naive B cells and memory B cells expressing one, two, or three Ig classes in five donors. **g** Proportions of single naive B cells and memory B cells expressing two Ig classes, including the combination of IgM and IgD, IgM, and IgG, IgG, and IgA and other observed combinations (IgM and IgA, IgM and IgE, IgD and IgG, IgD and IgA, IgD and IgE, IgG and IgE, and IgA and IgE)

It is well accepted that a single memory B or plasma cell post clonal expansion should display only one monoclonal V_H_DJ_H_ or V_L_J_L_ recombination pattern. To explore the characteristic V(D)J recombination patterns in B cells, we analyzed the Ig repertoire in B-cell subgroups. A 5′ GEX analysis of the fluorescence-activated cell sorting (FACS)-sorted CD19^+^ cells from healthy donors revealed diverse B-cell populations, including naive B, plasma, and memory B cells (Fig. 3a). Based on cell surface markers, cell clusters were categorized as follows: naive B cells with CD19^+^CD38^+/−^CD27^−^ and expressing immunoglobulin heavy constant μ chain (IGHM) and δ chain (IGHD), memory B cells with CD19^+^CD38^+/−^CD10^-^CD27^+^, and a small cluster of plasma cells with CD19^+^CD38^+++^. An analysis of immune repertoires showed that 5.40–7.71% of naive B cells and 7.00–10.68% of memory B cells expressed more than one V_H_DJ_H_ recombination patterns (Fig. 3b). Consistently, multiple V_L_J_L_ recombination patterns were also observed with an even higher frequency. Approximately, 11.38–14.74% of naive B cells and 9.28–14.16% of memory B cells exhibited two or three V_κ_J_κ_ recombination patterns (Fig. 3c). Moreover, 17.19–25.87% naive B cells and 12.42–18.03% memory B cells exhibited two or three V_λ_J_λ_ recombination patterns (Fig. 3d). In contrast, almost all plasma cells showed a single V_H_DJ_H_ and V_L_J_L_ recombination pattern (Fig. 3b–d). Co-expression of V_κ_J_κ_ and V_λ_J_λ_ recombinant transcripts was also observed in naive B cells (22.41–34.99%), plasma cells (12.59–28.18%) and memory B cells (8.70–15.69%) (Fig. 3e).

Analysis of 5′ GEX and VDJ repertoires showed that a much higher proportion of memory B cells (24.45–50.37%) than of naive B cells (7.38–36.87%) simultaneously expressed more than one classes of Ig transcripts (Fig. 3f). As expected, among all 754 naive B cells and 3571 memory B cells from five donors that expressed two classes of IgH chains, co-expression of Igμ and Igδ chains was mainly detected in naive B cells, but not in memory B cells. In contrast, co-expression of the Igα and Igγ chains mainly occurred in memory B cells, but not in naive B cells. However, co-expression of the Igμ and Igγ chains was identified, with a similar frequency, in either naive B cells or memory B cells, which is inconsistent with our previous understanding (Fig. 3g).

### Existence of multiple V(D)J recombination patterns in single B cells confirmed by Sanger sequencing

**Fig. 4:**
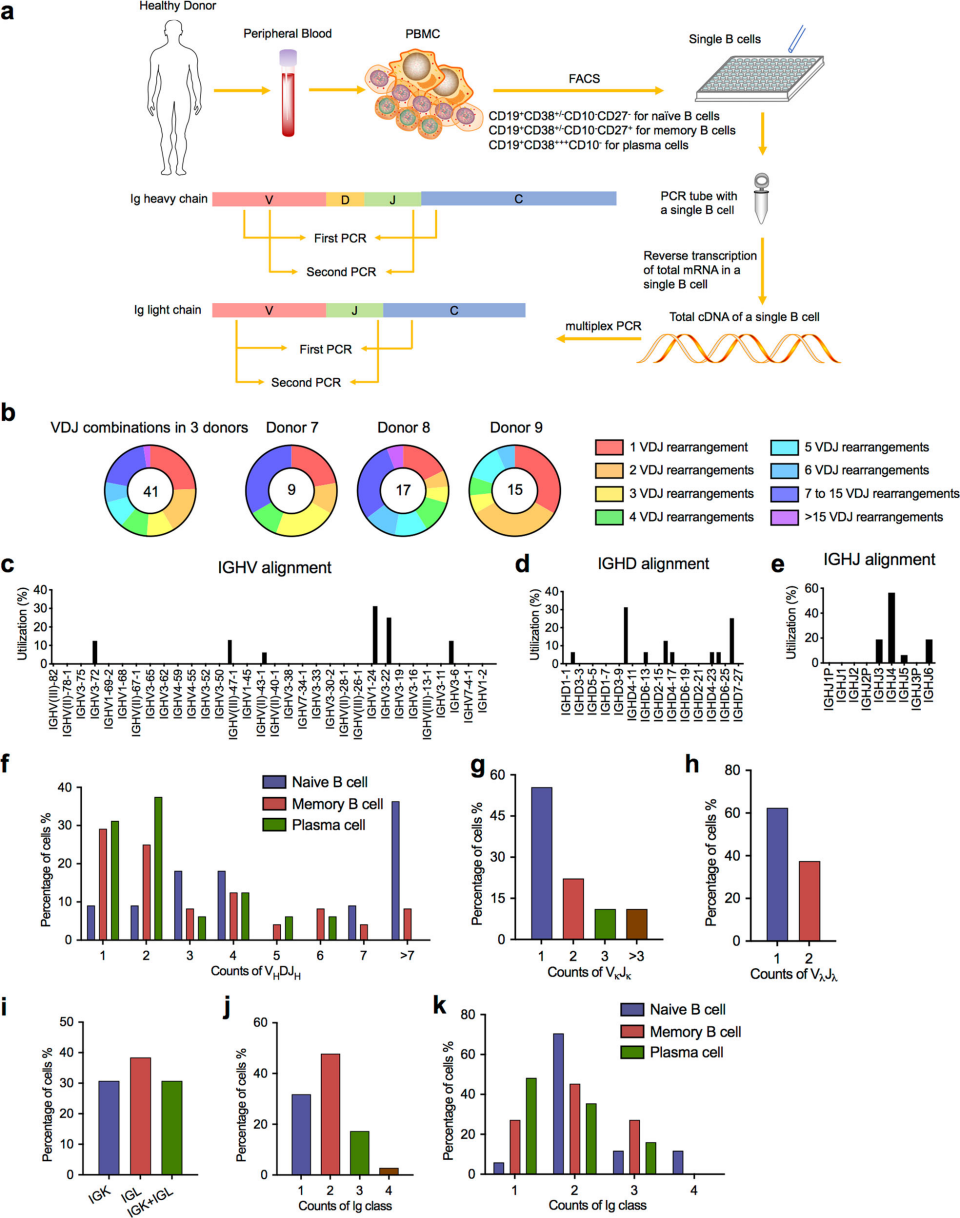
Sanger sequencing validation of multiple V(D)J recombination patterns in single B cells. **a** Sketching diagram of the single-cell sequencing procedure. We isolated peripheral blood from healthy donors and separated peripheral blood mononuclear cells (PBMCs). Naive B cells (CD19^+^CD38^+/−^CD10^-^CD27^−^), memory B cells (CD19^+^CD38^+/−^CD10^-^CD27^+^), and plasma cells (CD19^+^CD38^+++^CD10^−^) were sorted by FACS. The total mRNA in single B cells was reverse transcribed, and the Ig heavy chain and light chain were amplified by multiplex PCR. FACS, fluorescence-activated cell sorting. **b** Proportions of cells expressing VDJ recombination patterns in single B cells from donor 7, donor 8, and donor 9. **c**–**e** Distribution and frequency of IGHV (**c**), IGHD (**d**), and IGHJ (**e**) segments in Ig germline genes in a single cell. **f** Proportions of single naive B cells, plasma cells, and memory B cells expressing one, two, or three V_H_DJ_H_ segments. **g**, **h** Proportions of single B cells expressing one, two, or more V_κ_J_κ_ segments (**g**) and V_λ_J_λ_ segments (**h**). **i** Counts of single B cells expressing only Igκ or Igλ or expressing both Igκ and Igλ. **j**, **k** Proportions of naive B cells, plasma cells, and memory B cells expressing one, two, three, or four Ig classes

The discovery of two or more Ig VDJ transcripts in single B cells prompted us to dissect the Ig repertoires of single B cells by Sanger sequencing. CD19^+^ single B cells from the peripheral blood of additional four donors (donors 6–9) were sorted by flow cytometry. To avoid cross-contamination, each single cell was sorted individually into a 96-well plate and isolated by microtube anchoring under light microscopy. The total mRNA was subsequently extracted from the single B cells and reverse transcribed into cDNA following Tang’s protocol^28^. The IgV(D)J sequences were amplified with specific primers (Supplementary information, Table S8), and their repertoires of all five classes of IgH chains and two types of IgL chains were analyzed by multiple RT-PCRs and Sanger sequencing (Fig. 4a).

We first examined the Ig repertoires of eight single B cells from donor 6 and analyzed their Ig expression profiles. Consistent with the results of scRNA-seq, multiple recombination patterns of V_H_DJ_H_ or V_L_J_L_ were observed in the single B cells with different frequencies. Moreover, the presence of both Igγ and Igμ with different V_H_DJ_H_ recombination patterns was also detected in single B cells (Supplementary information, Table S9).

It is worth mentioning that the multiple rounds of cDNA amplification in both scRNA-seq and Tang’s method^28^ might induce differences in absolute copy number between high- and low-copy IgV(D)J transcripts, possibly causing low-copy Ig VDJ transcripts to be undetected due to the predominant amplification of high-copy transcripts. To address this issue, we compared the diversity of Ig VDJ transcripts between cDNAs with and without cDNA amplification. A total of 89 CD19^+^ single B cells FACS sorted from the peripheral blood of three healthy donors (donors 7–9) were isolated, and the mRNA from each single B cell was reverse transcribed into cDNA by a modified version of Tang’s method, not including the cDNA amplification step. The Ig repertoire of the cDNA from each single B cell was then analyzed by nested PCR and Sanger sequencing.

As expected, we observed more diverse IgV(D)J recombination patterns in the single B cells without cDNA amplification than in those that underwent cDNA amplification following Tang’s protocol. In total, we successfully captured the recombinant V_H_DJ_H_ transcripts of 41 single B cells and obtained 798 V_H_DJ_H_ patterns (Supplementary information, Table S10). Moreover, each single B-cell-derived Ig repertoire displayed unique VDJ rearrangement patterns, and no overlapping Ig repertoires was observed among the different single B cells. We also found that the majority of B cells (75.61%, 31/41) displayed two or more functional V_H_DJ_H_ recombination patterns, with some B cells (21.95%, 9/41) expressing more than seven V_H_DJ_H_ patterns (Fig. 4b). In contrast, only 24.39% (10/41) of single B cells displayed a monoclonal V_H_DJ_H_ pattern. These features were observed in all three donors, confirming the result that single B cells can express several patterns of V_H_DJ_H_ recombinant transcripts. It is known that only one V_H_DJ_H_ recombination pattern is retained after V_H_DJ_H_ rearrangement occurs by deleting the intervening V, D, and J segments located between the selected V and D or D and J segments from the genomic DNA. However, we detected IGHV (Fig. 4c), IGHD (Fig. 4d), and IGHJ (Fig. 4e) segments discontinuously distributed in the genome with different frequencies in an individual B cell. Multiple recombinant V_H_DJ_H_ transcripts were also identified in single naive B cells, memory B cells, and plasma cells (Fig. 4f).

We further analyzed the V–J recombinant transcripts of the κ chain or λ chain in 26 single B cells and identified 98 V–J recombination patterns of the κ chain and 77 of the λ chain. Specifically, each single B cell expressed its own unique Ig repertoire of V_κ_J_κ_ or V_λ_J_λ_ rearrangement, and some single B cells even expressed multiple V_κ_J_κ_ recombination patterns (44.44%, 4/9) (Fig. 4g) or transcripts of two V_λ_J_λ_ recombination patterns (32.50%, 3/8) (Fig. 4h). In contrast, most single B cells showed one unique V_κ_J_κ_ recombination pattern (69.2%, 18/26) or V_λ_J_λ_ recombination pattern (69.2%, 18/26), while the remaining single B cells (30.77%, 8/26) displayed V–J rearrangements with both the κ chain and λ chain (Fig. 4i), indicating that isotype exclusion of Ig light-chain genes was not established in these cells.

Similar to the results of scRNA-seq, most single B cells, including naive B cells, memory B cells, and plasma cells, could simultaneously express the transcripts of more than two classes of Ig transcripts (Fig. 4j, k), although the proportion of cells expressing one Ig class in memory B cells and plasma cells was higher than that in naive B cells. Each individual Ig class showed its own unique mono- or oligo-VDJ patterns without any shared VDJ patterns among different Ig classes in any single B cell (Supplementary information, Table S10).

## Discussion

Although antibodies (also known as immunoglobulins and Igs) have been known for over 100 years, the detailed mechanism of Ig production remains largely elusive. Among various hypotheses, including side-chain theory, template theory, and natural selection theory^6,29,30^, the theory of clonal selection^6^ is widely accepted based on the fundamental evidence that many immunocompetent cell clones exist in animals, and different clonal cells have different surface receptors capable of binding to the corresponding antigenic determinants^31,32^. Once an antigen binds to its corresponding clonal receptor, the cell clone is activated and produces a large number of Igs with the same specificity^33–35^. This theory is strongly supported by observations in malignant proliferating myeloma cells that can produce identically structured Igs and monoclonal antibody research^36^. Therefore, the theory that an antibody-producing cell produces only one antibody has been widely accepted. However, in the 1960s, some experimental evidence challenged this theory, as certain findings showed that two or even four different specific antibodies could be produced in a cell^37^. Through single B-cell-based sequencing analysis of the Ig repertoire, our results provide further supporting evidence that, at least in a group of B cells, clonal selection and classical CSR are not the only mechanism for antibody production, because multiple V(D)J rearrangements exist in many individual B cells, including naive B cells, memory B cells, and plasma cells, and different Ig classes in the same B cell do not share the same VDJ recombination.

The mechanism ensuring that a single B cell can produce only one V_H_DJ_H_ and one V_L_J_L_ recombination has been extensively studied. To date, three classical theories have been accepted. The first is the “allelic exclusion” theory, which states that Ig gene rearrangement occurs on one chromosome and is subsequently suppressed on the other chromosome^2^. In support of this hypothesis, two sizes of Ig heavy-chain variable region genes have been identified, including the small rearranged and the large unarranged Ig heavy chains^38,39^. The second is the “V(D)J rearrangement” theory, in which the intervening DNA segments between selected V_H_ and D_H_, D_H_ and J_H,_ or V_L_ and J_L_ segments are deleted to ensure that only one recombination pattern in both V_H_DJ_H_ and V_L_J_L_ occurs on a single chromosome^2,5,40^. The third is the “isotypic exclusion” theory, in which, once the V_κ_J_κ_ rearrangement occurs in the Igκ-type light-chain gene, the V_λ_J_λ_ rearrangement of the Igλ-type light-chain gene is suppressed^2,10^. This theory also explains why most mature B cells preferentially express κ chains and only a few B cells express λ chains. However, all three theories lack immune profiling evidence from individual B cells.

Through Chromium Single-Cell Immune Profiling of single CD19^+^ B cells from healthy donors, we demonstrated that a considerable proportion of single B cells displayed two or more V_H_DJ_H_ or V_κ_J_κ_ or V_λ_J_λ_ recombination patterns, even though most single B cells showed one V_H_DJ_H_ and one V_κ_J_κ_ or one V_λ_J_λ_ pattern. This finding is apparently inconsistent with the principle of “one B cell — one V(D)J copy” and suggests that, in many B cells, either the Ig gene segments between rearranged V and D or D and J segments are not deleted or the mechanism of “allelic exclusion” does not take effect. This hypothesis is substantiated by our findings that both transcripts of the κ chain and λ chain were frequently co-expressed in many single B cells. Through an optimized approach for unbiased detection of both high- and low-copy IgV(D)J transcripts, we further demonstrated the existence of multiple V_H_DJ_H_ or V_L_J_L_ recombination patterns in single B cells, and moreover, the proportion of B cells found to co-express the κ chain and λ chain was significantly increased. These results imply that it is a very common event that a single B cell expresses multiple V_H_DJ_H_ or V_L_J_L_ recombinant transcripts, and Ig diversity far exceeds initial estimates. It should be noted that our findings of “one B cell — multiple V(D)J copies” are mainly at the mRNA level, and whether these findings can be reproduced at the protein level remains unclear. We have hypothesized the following possible mechanisms. First, transposon mechanism may exist in some B cells, causing multiple VDJ recombination on an IgH locus. RAG1 is known to be evolved from transposase and still retains a transposase activity in mammalian cells although the efficiency of such reactions in vivo is lower comparing with its recombinase effect^41–43^. Second, incomplete DNA deletion may occur in some B cells, resulting in partial removal of the Ig gene segments between the selected D and J segments or between V and D segments, which allows multiple recombination on an IgH locus. Nevertheless, the underlying mechanisms for the occurrence of over two V(D)J exons or two Ig subclasses in a single B cell require more in-depth investigation at the genome level.

The classical concept explains that, to ensure “one single B cell — one antibody”, CSR does not change the VDJ usage of variable regions, and different Igs from the same B cell share the same V(D)J pattern^21^. However, our results clearly indicate that the mRNA transcripts of different IgH chains from the same single B cell do not share the same VDJ pattern. For example, IgM and IgD in a single B cell showed different V_H_DJ_H_ recombination patterns producing IgM and IgD with different antigen specificities. The results are beyond the current understanding of immunological knowledge and suggest that at least some single B cells have different specificities, which can recognizes different antigens. Here, we proposed a potential possibility that during the early-stage of contact with antigen, monoclonal, or oligoclonal naive B cells expressing specific IgM and IgD are primarily screened out and secreting low-affinity IgM, which is responsible for early humoral immune response. Subsequently, following selection with the antigen, the other B-cell subsets with specific IgG, IgA, or IgE are activated and induced to secrete highly specific IgG, IgA, or IgE with high affinity and responsible for later-stage humoral immune response.

Generally, upon encountering antigens, only a few naive B cells can be clonally selected, expanded and differentiated into plasma cells or memory B cells; therefore, single memory B cells or plasma cells should secrete only an Ig class with monoclonal features. However, in this study, another result exceeding our expectations was that multiple V_H_DJ_H_ or V_L_J_L_ recombinant transcripts and multiple Ig classes were frequently found in memory B cells. Therefore, moving beyond the classical theories of “one single B cell — one antibody”, our findings led us to reasonably speculate that a single B cell expressing multiple B-cell receptors (BCRs) can rapidly capture antigens and, through multiple low-affinity BCRs, induce the expansion of a number of specific B cells via pattern recognition. However, one B cell producing only one specific antibody would be unlikely to rapidly find its matching antigen. In addition, it is more reasonable that different Ig classes genetically express their own Ig repertoires compared with the classical model of CSR.

Consistently, our reanalysis of the raw data released by Neal et al.^26^ also revealed the existence of multi-V(D)J patterns in many single B cells. In summary, our data demonstrate novel patterns of Ig gene rearrangement and class switching, which are worthy of further investigation to clarify how a single B cell produces multiple distinct Igs.

## Materials and methods

### Isolation and preparation of mononuclear cells from peripheral blood

Peripheral blood was obtained from nine healthy donors. Mononuclear cells were isolated from 5 mL of peripheral blood using Percoll. The white gradient layer containing PBMCs was isolated and washed with phosphate buffer solution (PBS). The isolated PBMCs were used for sorting B cells. This study was approved by the Institutional Review Board of Peking University (PU IRB).

### Sorting single B cells from subsets

To sort the subsets of B cells, the isolated PBMCs were washed in PBS, blocked with 5% fetal bovine serum (FBS) for 30 min on ice, and then stained with mouse anti-human CD19 APC (BioLegend, 302211), mouse anti-human CD10 PerCP-eFluor (eBioscience, 46-0108-41), mouse anti-human CD27 FITC (eBioscience, 11-0279-41), mouse anti-human CD38 PE/Cy7 (eBioscience, 25-0388-41), and mouse anti-human IgM APC/Cy7 (BioLegend, 314519) for 30 min on ice in the dark. The stained cells were washed in PBS, and single cells were sorted by flow cytometry. Naive B cells were defined as CD38^+/−^CD10^−^ cells gated on CD19^+^ cells. Memory B cells were defined as CD38^++^CD10^+^ cells gated on CD19^+^ cells. Plasma cells were defined as CD38^+++^CD10^−^ cells gated on CD19^+^ cells. The B cells were sorted into 96-well plates by flow cytometric sorting to ensure that one cell was sorted into each well and avoid contamination. Each single B cell was carefully removed under a microscope with a mouth pipette and collected separately.

### 10× library preparation and sequencing

The concentration of the single-cell suspension was counted using Invitrogen™ Countess™ II FL (Thermo) and adjusted to 1000 cells/μL. Cells were loaded according to a standard protocol to capture 7000 cells/chip position. All remaining procedures, including library construction, were performed according to the manufacturer’s standard protocol. Single-cell cDNA was separated into two aliquots for scRNA-seq library and V(D)J-enriched library generation from the same input sample. scRNA-seq libraries were prepared using the Chromium Single Cell 5′ Library Construction Kit (10× Genomics, 1000020), and full-length V(D)J segments were enriched from the amplified cDNA with primers specific to the BCR regions using the Chromium Single Cell V(D)J Enrichment Kit (10× Genomics, 1000016). The libraries were sequenced on an Illumina HiSeq X-Ten platform using 150 nt paired-end sequencing.

### BCR mapping

The Chromium 10× V(D)J single-cell sequencing data were mapped against the human (hg38) reference genome (released by 10× with VDJ information) with Cell Ranger (v3.0.2)^44^. The results of the filtered annotation file for the five samples were then used to perform the downstream calculations with custom scripts.

### scRNA-seq data analysis

The raw scRNA-seq data were mapped against the human (hg38) reference genome using Cell Ranger (v3.0.2)^44^ with the default parameters. Then, the Seurat package (v3.0.0)^45^ was employed for downstream analysis. To filter out low-quality cells, stringent quality filter criteria were applied to each cell (nFeature_RNA > 200; nFeature_RNA < 3500; percent.mt < 10%). Then, the data were log normalized with a multiplied scale factor of 10,000. Subsequently, the vst method was used to identify the variable features, and 2000 highly variable genes were selected. Canonical correlation analysis (CCA) was used to remove the batch effects among the five samples with dimensions = 20. The highly variable genes in the scaled data were subjected to principal component analysis. The first 20 principal components were used to cluster the cells and perform a subtype analysis by nonlinear dimensional reduction (t-SNE). Classification of B-cell subgroups was inferred from the annotation of cluster-specific genes; naive B cells (CD19^+^CD38^+/−^CD27^−^IGHM^+^IGHD^+^); memory B cells (CD19^+^CD38^+/−^CD27^+/−^ with high Ig expression); plasma cells (CD19^+^CD38^+++^CD27^+^ with high Ig expression).

To link the 5′ RNA-seq data to the VDJ data, the barcodes between the B cell 5′ GEX samples overlapped with those of the VDJ B enrichment libraries. There was a high fraction of barcode overlap between the GEX and VDJ libraries. In total, 5567 of 5901 cells detected by VDJ B enrichment assay were also detected by 5′ GEX in donor 1, representing a 94.34% overlap between the GEX and VDJ B enrichment libraries. Similarly, the barcode overlaps between the 5′ GEX and VDJ B enrichment libraries were 89.94% (3360/3736) in donor 2, 86.49% (3846/4447) in donor 3, 87.27% (2420/2773) in donor 4, and 87.29% (3751/4297) in donor 5.

### Extraction and reverse transcription of RNA in single cells

The protocol of single-cell lysis and reverse transcription of total RNA was performed as described in Tang’s masterpiece^28^. In brief, a single B cell was first isolated and put into lysate buffer by mouth pipette. The total mRNA was extracted from single B cells, and reverse transcription was then performed directly on the whole-cell lysate. After this, the free primers were removed by Exonuclease I, and a poly(A) tail was added to the 3′ end of the first-strand cDNA by Terminal Deoxynucleotidyl Transferase (TdT) (Invitrogen, cat. no. 10533-065) for cDNA amplification. Then, the single-cell cDNA was amplified by 20 plus five cycles of PCR. The amplified cDNA of a single B cell was used to amplify Igs by nested PCR. To acquire single-cell cDNA without amplification, the step involving addition of a poly(A) tail by TdT was removed, while the other steps remained.

### Nested PCR

Part of the variable region of the IgH gene was amplified by two rounds of nested PCR. The first round was carried out with the upstream primers VH1-FR1, VH2-FR1, VH3-FR1, VH4-FR1, VH5-FR1, and VH6-FR1 and the downstream primers IGHG-R1, IGHA-R1, IGHM-R1, IGHD-R1, and IGHE-R1, respectively, to amplify Igγ, Igα, Igμ, Igδ, and Igε. The second round was carried out with the upstream primer VH and the downstream primer JH to amplify the variable region of IgH. The variable region of the Igκ gene was amplified by half-nested PCR. The first round was carried out with the upstream primers VK1F/6, VK2F, VK3F, VK4F, VK5F, and VK7F and the downstream primer IGKC-R3. The second round used the same upstream primers and the downstream primer IGKC-R1. The variable region of the Igλ gene was also amplified by half-nested PCR. The first round was carried out with the upstream primers VL1/2F and VL3F and the downstream primer IGLC-R3. The second round used the same upstream primers and the downstream primer JL. Touchdown PCR was performed for amplification. The sequences of all primers used are listed in Supplementary Information, Table S8.

### Sanger sequencing

PCR products were cloned into the pGEM-T Easy vector (Promega, Madison, WI) and sequenced with an ABI 3100 Genetic Analyzer (Applied Biosystems, Foster City, CA). The V(D)J sequences were compared with those found in the BLAST search to identify the best matching germline gene segments and V(D)J junctions.

## Supplementary information

Supplementary Information

Table S1

Table S2

Table S3

Table S4

Table S5

Table S6

Table S7

Table S8

Table S9

Table S10

## Data Availability

The accession number for the sequencing data in this paper is CRA001862. These data have been deposited in the Genome Sequence Archive under project PRJCA001617.

## Supplementary information

**Supplementary information** accompanies the paper at (10.1038/s41421-019-0137-3).
