# Supplementary material for: More than one antibody of individual B cells revealed by single-cell immune profiling
Source: Cell Discov. 2019 Dec 10;5:64. doi: 10.1038/s41421-019-0137-3 (PMC6901605; doi:10.1038/s41421-019-0137-3)
Supplement: Supplementary file 1 — Supplementary Information [file 41421_2019_137_MOESM1_ESM.pdf]

Supplementary information, Fig. S1

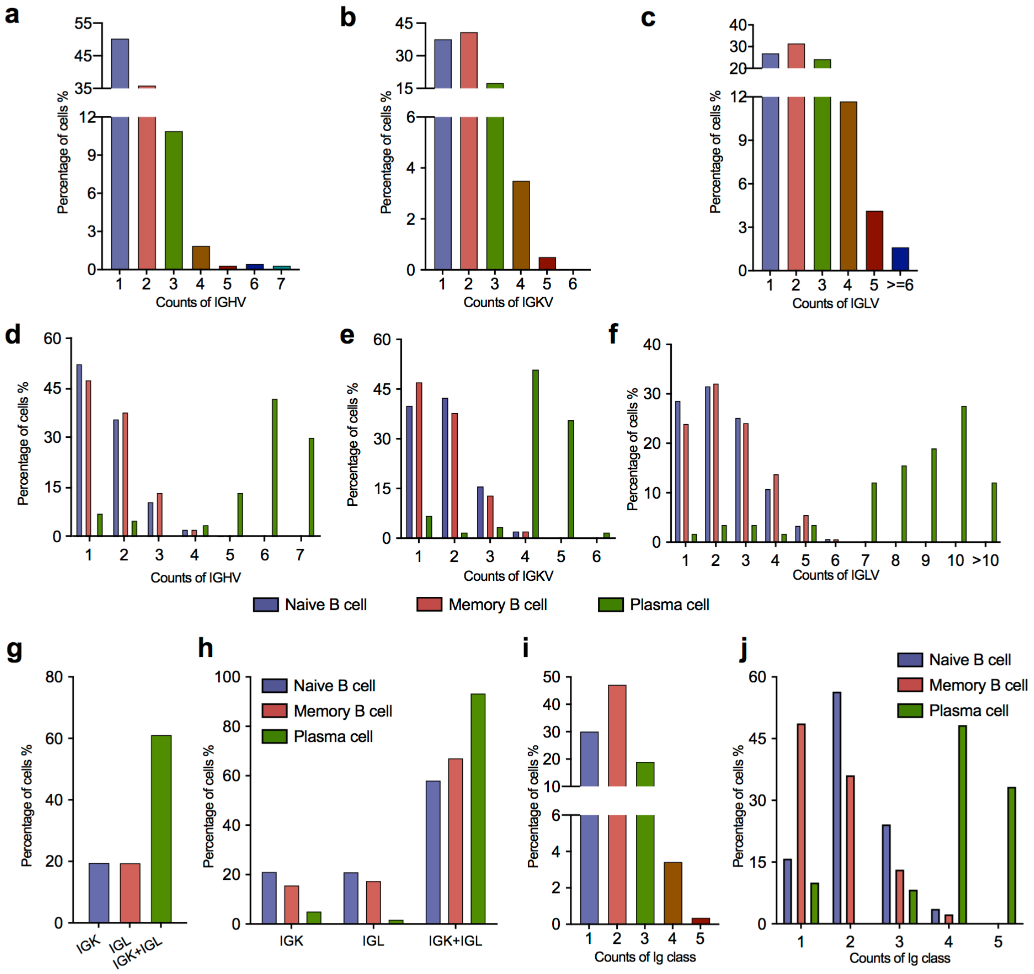

Supplementary Fig. S1 Ig repertoires of V(D)J-seq before assembly in single B cells of donor 1

(**a-c**) Proportions of single PBMCs expressing more than one IGHV (**a**), IGKV (**b**) and IGLV (**c**) segment from donor 1.

(**d-f**) Proportions of single naïve B cells, memory B cells and plasma cells (**c**) expressing one, two or three IGHV segments (**d**), IGKV segments (**e**) and IGLV segments (**f**) in donor 1.

(**g**) Proportions of single B cells expressing only Igκ or Igλ or expressing Igκ and Igλ in donor 1.

(**h**) Proportions of single naïve B cells, plasma cells and memory B cells expressing only Igκ or Igλ or expressing Igκ and Igλ in donor 1.

(**i**) Proportions of single naïve B cells and memory B cells expressing one, two or three Ig classes in donor 1.

(**j**) Proportions of single naïve B cells, memory B cells and plasma cells expressing two Ig classes, including the combination of IgM and IgD, IgM and IgG, IgG and IgA and other observed combinations (IgM and IgA, IgM and IgE, IgD and IgG, IgD and IgA, IgD and IgE, IgG and IgE and IgA and IgE).

Supplementary information, Fig. S2

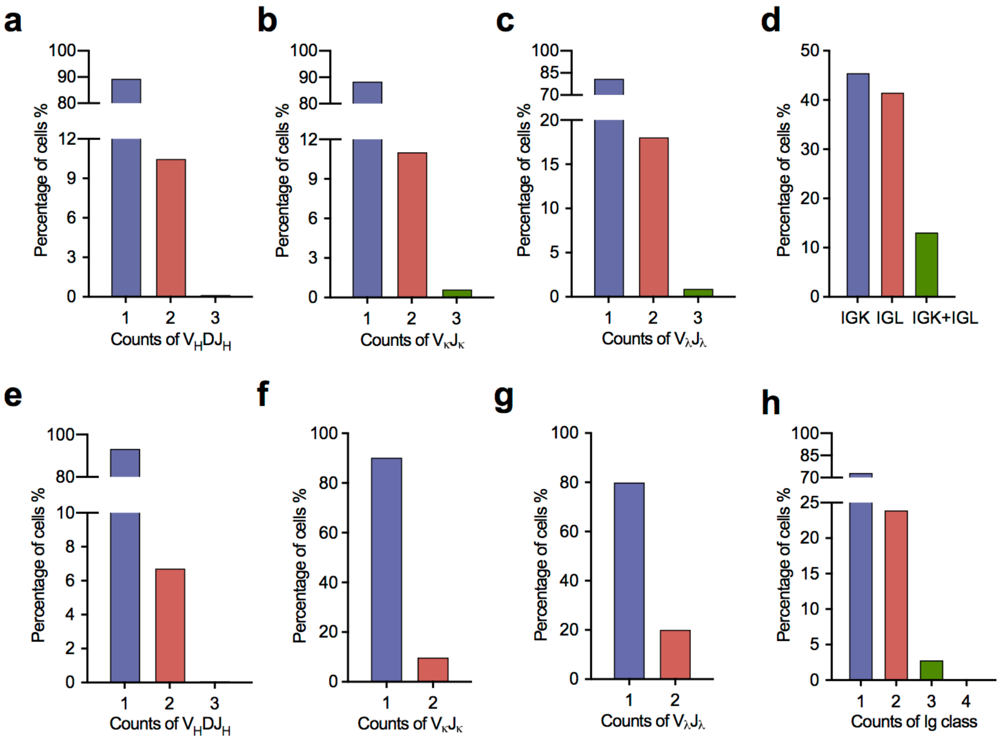

Fig. S2 Ig repertoires of single B cells retrieved from two 5' RNA-seq and V(D)J-seq databases

(**a-c**) Proportions of single B cells expressing more than one V_HDJ_H (**a**), $V_\kappa J_\kappa$ (**b**) and $V_\lambda J_\lambda$ (**c**) segment.

(**d**) Proportions of single B cells expressing only Igκ or Igλ or expressing both Igκ and Igλ.

(**e-g**) Proportions of single PBMCs expressing more than one V_HDJ_H (**f**), $V_\kappa J_\kappa$ (**g**) and $V_\lambda J_\lambda$ (**g**) segment.

(**h**) Proportions of single cells expressing one, two, three or four Ig classes.

a-d, h, data from the GEO database. GEO: GSE111360. **e-g**, data from the single-cell data website of 10× Genomics, available at https://support.10xgenomics.com/single-cell-vdj/datasets/2.2.0/vdj_v1_hs_pbmc_b.

Supplemental Tables

Supplementary information, Table S1. V(D)J recombination patterns and Ig sequences in single B cells of donor 1, Related to Figures 1, 2 and Figure S1

Supplementary information, Table S2. V(D)J recombination patterns and Ig sequences in single B cells of donor 2, Related to Figures 1, 2 and Figure S1

Supplementary information, Table S3. V(D)J recombination patterns and Ig sequences in single B cells of donor 3, Related to Figures 1, 2 and Figure S1

Supplementary information, Table S4. V(D)J recombination patterns and Ig sequences in single B cells of donor 4, Related to Figures 1, 2 and Figure S1

Supplementary information, Table S5. V(D)J recombination patterns and Ig sequences in single B cells of donor 5, Related to Figures 1, 2 and Figure S1

Supplementary information, Table S6. V(D)J recombination patterns and Ig sequences in single B cells from healthy donor PBMCs retrieved from GEO database (GEO: GSE111360), Related to Figure S2a-d, h

Supplementary information, Table S7. V(D)J recombination patterns and Ig sequences in single B cells retrieved from the database of 10× Genomics, Related to Figure S2e-g

Supplementary information, Table S8. Specific primers against immunoglobulins used in this study, Related to Figure 4

Supplementary information, Table S9. V(D)J recombination patterns and Ig sequences in single B cells of donor 6, Related to Figure 4

Supplementary information, Table S10. V(D)J recombination patterns and Ig sequences in single B cells of donors 7-9, Related to Figure 4
